# Imputation-Based Analysis of Association Studies: Candidate Regions and Quantitative Traits

**DOI:** 10.1371/journal.pgen.0030114

**Published:** 2007-07-27

**Authors:** Bertrand Servin, Matthew Stephens

**Affiliations:** Department of Statistics, University of Washington, Seattle, Washington, United States of America; University of Alabama at Birmingham, United States of America

## Abstract

We introduce a new framework for the analysis of association studies, designed to allow untyped variants to be more effectively and directly tested for association with a phenotype. The idea is to combine knowledge on patterns of correlation among SNPs (e.g., from the International HapMap project or resequencing data in a candidate region of interest) with genotype data at tag SNPs collected on a phenotyped study sample, to estimate (“impute”) unmeasured genotypes, and then assess association between the phenotype and these estimated genotypes. Compared with standard single-SNP tests, this approach results in increased power to detect association, even in cases in which the causal variant is typed, with the greatest gain occurring when multiple causal variants are present. It also provides more interpretable explanations for observed associations, including assessing, for each SNP, the strength of the evidence that it (rather than another correlated SNP) is causal. Although we focus on association studies with quantitative phenotype and a relatively restricted region (e.g., a candidate gene), the framework is applicable and computationally practical for whole genome association studies. Methods described here are implemented in a software package, Bim-Bam, available from the Stephens Lab website http://stephenslab.uchicago.edu/software.html.

## Author Summary

Ongoing association studies are evaluating the influence of genetic variation on phenotypes of interest (hereditary traits and susceptibility to disease) in large patient samples. However, although genotyping is relatively cheap, most association studies genotype only a small proportion of SNPs in the region of study, with many SNPs remaining untyped. Here, we present methods for assessing whether these untyped SNPs are associated with the phenotype of interest. The methods exploit information on patterns of multi-marker correlation (“linkage disequilibrium”) from publically available databases, such as the International HapMap project or the SeattleSNPs resequencing studies, to estimate (“impute”) patient genotypes at untyped SNPs, and assess the estimated genotypes for association with phenotype. We show that, particularly for common causal variants, these methods are highly effective. Compared with standard methods, they provide both greater power to detect associations between genetic variation and phenotypes, and also better explanations of detected associations, in many cases closely approximating results that would have been obtained by genotyping all SNPs.

## Introduction

Although the development of cheap high-throughput genotyping assays have made large-scale association studies a reality, most ongoing association studies genotype only a small proportion of SNPs in the region of study (be that the whole genome, or a set of candidate regions). Because of correlation (linkage disequilibrium, LD) among nearby markers, many untyped SNPs in a region will be highly correlated with one or more nearby typed SNPs. Thus, intuitively, testing typed SNPs for association with a phenotype will also have some power to pick up associations between the phenotype and untyped SNPs. In practice, typical analyses involve testing each typed SNP individually, and in some cases combinations of typed SNPs jointly (e.g., haplotypes), for association with phenotype, and hoping that these tests will indirectly pick up associations due to untyped SNPs. Here, we present a framework for more directly and effectively interrogating untyped variation.

In outline, our approach improves on standard analyses by exploiting available information on LD among untyped and typed SNPs. Partial information on this is generally available from the International HapMap project [1]; in some cases more detailed information (e.g., resequencing data) may also be available, either through public databases (e.g., SeattleSNPs [2]), or through data collected as a part of the association study design (e.g., [3]). Our approach combines this background knowledge of LD with genotypes collected at typed SNPs in the association study, to explicitly predict (“impute”) genotypes in the study sample at untyped SNPs, and then tests for association between imputed genotypes and phenotype. We use statistical models for multi-marker LD to perform the genotype imputation, with uncertainty, and a Bayesian regression approach to perform the test for association, allowing for potential errors in the imputed genotypes. Although we focus specifically on methods for analyzing quantitative phenotypes in candidate gene studies, the same general framework can also be applied to discrete traits, and/or genome-wide scans.

These imputation-based methods can be viewed as a natural *analysis* complement to the “tag SNP” *design* strategy for association studies, which attempts to choose SNPs that are highly correlated with, and hence good predictors of, untyped SNPs. We are simply directly exploiting this property, together with recently developed statistical models for multi-locus LD ([4,5]) to infer the untyped SNP genotypes. Our approach is also somewhat analogous to multipoint approaches to linkage mapping (e.g., [6]), in which observed genotypes at multiple markers predict patterns of identity by descent (IBD) at nearby positions without markers, and test for correlation between these patterns of IBD and observed phenotypes. In the association context, we are predicting identity by state rather than IBD, and the methods of predicting identity by state versus IBD differ greatly, but the approaches share the idea of using multipoint information to predict single-point information, and, at least in their simplest form, subsequently assessing correlation with phenotype at the single-point level. This strategy provides a clean and rigorous way to avoid the “curse of dimensionality” that can plague haplotype-based analyses, without making *ad hoc* decisions such as pooling rare haplotypes into a single class.

Although our methods are developed in a Bayesian framework, they can also be used to compute *p-*values assessing significance of observed genotype–phenotype associations. Our approach should therefore be of interest to practitioners whether or not they favor Bayesian procedures in general. It has two main advantages over more standard approaches. First, it provides greater power to *detect* associations. Part of this increased power comes from incorporating extra information (knowledge on patterns of LD among typed and untyped SNPs), but, unexpectedly, we also found an increased power of our Bayesian approach even when all SNPs were actually typed. Second, and perhaps more importantly, it provides *more interpretable explanations* for potential associations. Specifically, for each SNP (typed and untyped), it provides a probability that it is causal. This contrasts with standard single-SNP tests, which provide a *p-*value for each SNP, but no clear way to decide which SNPs with small *p-*values might be causal.

## Methods

We focus on an association study design in which genotype data are available for a dense set of SNPs on a panel of individuals, and genotypes are available for a subset of these SNPs (which for convenience we refer to as “tag SNPs”) on a cohort of individuals who have been phenotyped for a univariate quantitative trait. We assume the cohort to be a random sample from the population, and consider application to other designs in the discussion.

Our strategy is to use patterns of LD in the panel, together with the tag SNP genotypes in the cohort, to explicitly predict the genotypes at all markers for members of the cohort, and then analyze the data *as if the cohort had been genotyped at all markers*. There are thus two components to our approach: (i) predicting (“imputing”) cohort genotypes, and (ii) analyzing association between cohort genotypes and phenotypes. For (i), we use existing models for population genetic variation across multiple markers [4,5], which perform well at estimating missing genotypes, and provide a quantitative assessment of the uncertainty in these estimates [5]. For (ii), we introduce a new approach based on Bayesian regression, and describe how this approach can yield not only standard Bayesian inference, but also *p-*values for testing the null hypothesis of no genotype–phenotype association. We chose to take a Bayesian approach partly because it provides a natural way to consider uncertainty in estimated genotypes. However, the Bayesian approach has other advantages; in particular, it provides a measure of the strength of the evidence for an association (the Bayes factor, BF) that is, in some respects, superior to conventional *p-*values. Furthermore, in our simulations, *p-*values from our Bayesian approach provide more powerful tests than standard tests, even if the cohort is actually genotyped at *all* markers (including all causal variants).

### Bayesian Regression Approach

We now provide further details of our Bayesian regression approach. The literature on Bayesian regression methods is too large to review here, but papers particularly relevant to our work include [7–9].

For simplicity, we focus on the situation where cohort genotypes are known at all SNPs (tag and non-tag). Extension to the situation, where the cohort is genotyped only at tag SNPs and other genotypes are imputed using sampling-based algorithms such as PHASE [10,11] or fastPHASE [5], is relatively straightforward (see below).

Let G denote the cohort genotypes for all *n* individuals in the cohort, and *y =* (*y*_1_, …, *y_n_*) denote the corresponding (univariate, quantitative) phenotypes. We model the phenotypes by a standard linear regression:

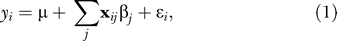
where *y_i_* is the phenotype measurement for individual *i*, μ is the phenotype mean of individuals carrying the “reference” genotype, the *x_ij_*s are the elements of a design matrix *X* (which depends on the genotype data; see below), the β*_j_*s are the corresponding regression coefficients, and ɛ*_i_* is a residual. We assume ɛ*_i_*s are independent and identically distributed ∼*N*(0,1/τ), where τ denotes the inverse of the variance, usually referred to as the *precision* (we choose this parameterization to simplify notation in later derivations). Thus 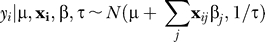 and:

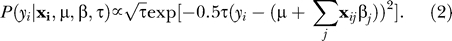



We assume a genetic model where the genetic effect is additive across SNPs (i.e., no interactions) and where the three possible genotypes at each SNP (major allele homozygote, heterozygote, and minor allele homozygote) have effects 0, *a* + *ak* and 2*a,* respectively [12]. We achieve this by including two columns in the design matrix for each SNP, one column being the genotypes (coded as 0, 1, or 2 copies of the minor allele), and the other being indicators (0 or 1) for whether the genotype is heterozygous. The effect of SNP *j* is then determined by a pair of regression coefficients (β*_j1_,* β*_j_*_2_), which are, respectively, the SNP additive effect *a_j_* and dominance effect *d_j_ = a_j_k_j_*. While there are other ways to code the correspondence between genotypes and the design matrix, we chose this coding to aid specifying sensible priors (see below).

#### Priors for (**β**, **μ**, **τ**).

Prior specification is intrinsically subjective, and specifying priors that satisfy everyone is probably a hopeless goal. Our aim is to specify “useful” priors, which avoid some potential pitfalls (discussed below), facilitate computation, and have some appealing properties, while leaving some room for context-specific subjective input. In particular, we describe two priors below, which we refer to as prior *D_1_* and *D*_2_, that were developed based on the following considerations: (i) inference should not depend on the units in which the phenotype is measured; (ii) even if the phenotype is affected by SNPs in this region, the majority of SNPs will likely not be causal; (iii) for each causal variant there should be some allowance for deviations from additive effects (i.e., dominant/recessive effects) without entirely discarding additivity as a helpful parsimonious assumption; and (iv) computations should be sufficiently rapid to make application to genome-wide studies practical (this last consideration refers to prior *D*_2_).

#### Priors on the phenotype mean and variance.

The parameters μ and τ relate to the mean and variance of the phenotype, which depend on units of measurement. It seems desirable that estimates (and, more generally, posterior distributions) of these parameters scale appropriately with the units of measurement, so, for example, multiplying all phenotypes by 1,000 should also multiply estimates of μ by 1,000. Motivated by this, for prior *D*_1_ we used Jeffreys' prior for these parameters:

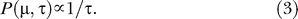



This prior is well known to have the desired scaling properties in the simpler context where observed data are assumed to be *N*(μ, 1/τ) [13], and we conjecture that our prior *D*_1_ also possesses these desired scaling properties in the more complex context considered here, although we have not proven this.

For prior *D*_2_ we used a slightly different prior, based on assuming a prior for (μ, τ) of the form

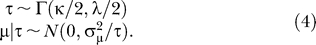



Specifically, our prior *D*_2_ assumes the limiting form of this prior as κ,λ → 0 and 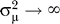 . In Protocol S1 we show that the posterior distributions obtained using this limiting prior scale appropriately.

Both prior distributions above are “improper” (meaning that the densities do not integrate to a finite value). Great care is necessary before using improper priors, particularly where one intends to compute BFs to compare models, as we do here. However, we believe results obtained using these priors are sensible. For prior *D*_2_ *,* as we show in Protocol S1 the posteriors are proper, and the BF tends to a sensible limit. For prior *D*_1_ we believe this to be true, although we have not proven it.

#### Prior on SNP effects.

For brevity, we refer to SNPs that affect phenotype as QTNs, for quantitative trait nucleotides. Our prior on the SNP effects has two components: a prior on which SNPs are QTNs and a prior on the QTN effect sizes.

#### Prior on which SNPs are QTNs.

We assume that with some probability, *p*_0_, none of the SNPs is a QTN; that is, the “null model” of no genotype–phenotype association holds. Otherwise, with probability (1 − *p*_0_), we assume there are *l* QTNs, where *l* has some distribution *p*(*l*) on {1, 2, …, *n_s_*} where *n_s_* denotes the number of SNPs in the region. Given *l*, we assume all subsets of *l* SNPs are equally likely. Both *p*_0_ and *p*(*l*) can be context-dependent, and choice of appropriate values is discussed below.

#### Prior on effect sizes.

If SNP *j* is a QTN, then its effect is modeled by two parameters, *a_j_* and *d_j_ = a_j_k_j_*. The parameter *a_j_* measures a deviation from the mean μ and will depend on the unit of measurement of the phenotype. To reflect this, we scale the prior on *a_j_* by the phenotypic standard deviation within each genotype class, 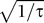 . Specifically, our prior on *a_j_* is 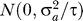 , where σ*_a_* reflects the typical size of a QTN effect compared with the phenotype standard deviation within each genotypic class. Choice of σ*_a_* may be context-dependent, and is discussed below.

The parameter *d_j_ = a_j_k_j_* measures the dominance effect of a QTN. If *k_j_ =* 0, then the QTN is additive: the heterozygote mean is exactly between the means of the two homozygotes. If *k_j_ =* 1 (respectively, −1), allele 1 (respectively, 0) is dominant. The case 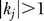 corresponds to overdominance of allele 1 or allele 0. We investigate two different priors for the dominance effect:

Prior *D*_1_ **:** We assume that *k_j_* is a priori independent of *a_j_*, with 
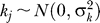
 . We chose σ*_k_* = 0.5, which gives 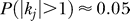 , reflecting a belief that overdominance is relatively rare.

Prior *D*_2_: We assume that *d_j_* is a priori independent of *a_j_*, with 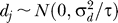 , where we took σ*_d_* = 0.5σ*_a_*. This prior on *d_j_* induces a prior on *k_j_* in which *k_j_* is not independent of *a_j_*.

Prior *D*_1_ has the attractive property that the prior probability of overdominance is independent of the QTN additive effect *a_j_*. However, the posterior distributions of *a_j_* and *k_j_* must be estimated via a computationally intensive Markov Chain Monte Carlo (MCMC) scheme (see Protocol S2). (An alternative, which we have not yet pursued, would be to approximate BFs under prior *D*_1_ by numerical methods, such as Laplace Approximation; e.g., [14]). Prior *D*_2_ is more convenient, as, when combined with the priors on μ and τ in Equation 4, posterior probabilities of interest can be computed analytically (Protocol S1).

For both priors *D*_1_ and *D*_2_ we assume effect parameters for different SNPs are, a priori, independent (given the other parameters).

#### Choice of *p*_0_, *p*(*l*), and σ*_a_*.

The above priors include “hyperparameters,” *p*_0_ and σ*_a_*, and a distribution *p*(*l*) that must be specified. The hyperparameter *p*_0_ gives the prior probability that the region contains no QTNs. While choice of appropriate value is both subjective and context-specific, for candidate regions we suggest *p*_0_ will typically fall in the range 10^−2^ to 0.5. If data on multiple regions are available, then it might be possible to estimate *p*_0_ from the data, although we do not pursue this here. Instead, we mostly sidestep the issue of specifying *p*_0_ by focusing on the BF (described below), which allows readers of an analysis to use their own value for *p*_0_ when interpreting results.

In specifying the prior, *p*(*l*), for the number of QTNs, we suggest concentrating most of the mass on models with relatively few QTNs. Indeed, here we focus mainly on the extreme case in which *p*(*l*) is entirely concentrated on *l* = 1: that is, the “alternative” model is that the region contains a single QTN. Although rather restrictive, this seems a good starting point in practice, particularly since our results show that it can perform well even if multiple QTNs are present. Nonetheless, there are advantages to considering models with multiple QTNs, and so we also consider a prior where *p*(*l*) puts equal mass on *l* = 1, 2, 3, or 4. This prior suffices to illustrate the potential of our approach, although in practice it would probably be preferable to place decreasing probabilities on larger numbers of QTNs (e.g., *p*(*l* = 2) < p(*l* = 1)). An alternative would be to sidestep specifying *p*(*l*) by computing BFs comparing, say, 4-QTN, 3-QTN, 2-QTN, and 1-QTN models versus the “null” model. However, interpreting and acting on these BFs will inevitably correspond to implicit assumptions about the relative prior plausibility of these multi-QTN models.

Finally, specification of the standard deviation of the effect size, σ*_a_*, involves subtle issues. Although it may seem tempting to use “large” σ*_a_* to reflect relative “ignorance” about effect sizes [15], we believe this is inadvisable. Although large σ*_a_* yields a flat prior on effect sizes, this prior is far from uninformative, in that it places almost all its mass on large effect sizes. The result would essentially allow only zero effects (i.e., the “null” model), or large effects (the “alternative” model). If in truth the causal SNPs have relatively small effect, which is probably generally realistic, then (for realistic sample sizes) the null model would be strongly favored over the alternative, because the data would be more consistent with zero effects than with large effects. Choice of σ*_a_* can thus strongly affect inference, particularly the BF, which we use to summarize evidence for the region containing any QTNs. Partly because of this, in practice we suggest averaging results over several values for σ*_a_* (equivalent to placing a prior on σ*_a_*). It may also be helpful to examine sensitivity of results to σ*_a_*. For example, if the BF is small for all values of σ*_a_,* then there is no evidence for any QTN in the region; if the BF is large for some values and small for others, then the evidence depends on the extent to which you believe in large versus small effects. However, for simplicity, all results in this paper were obtained using a fixed value of σ*_a_* = 0.5.

### Inference

We focus on two key inferential problems: (i) *detecting* association between genotypes and phenotype, and (ii) *explaining* observed associations. In the model of Equation 1, these translate to answering (i) are any β*_j_*s non-zero? and (ii) which β*_j_*s are non-zero and how big are they? We view the ability to address both questions within a single framework to be an advantage of our approach.

#### Detecting association.

To measure the evidence for *any* association between genotypes and phenotypes, we use the BF, [16] given by

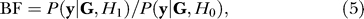
where *H*_0_ denotes the null hypothesis that none of the SNPs is a QTN (*a_j_ = d_j_ =* 0 for all *j*), and *H*_1_ denotes the complementary event (i.e., at least one SNP is a QTN). Computing the BF involves integrating out unknown parameters, as described in Protocols S1 and S2. In interpreting a BF, it is helpful to bear in mind the formula “posterior odds = prior odds × BF,” so, for example, if the prior odds are 1:1 (i.e., *p*_0_ = 0.5, so association with genetic variation in the region is considered equally plausible, a priori, as no association) then a BF of 10 gives posterior odds of 10:1, or ∼91% probability of an association.

In the special case where we allow at most one QTN, Equation 5 reduces to

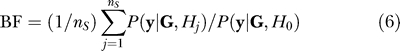
where *H_j_* denotes the event that SNP *j* is the QTN. The *j*th term in this sum corresponds to the BF for *H_j_* versus the null model, and involves the genotype data at SNP *j* only. We refer to these terms as the “single-SNP” BFs, so in this special case the overall BF is the mean of the single-SNP BFs. This natural way for combining information across (potentially correlated) SNPs is an attractive property of BFs compared with single-SNP *p-*values. Furthermore, in terms of detecting a genotype–phenotype association it can work well even if multiple QTNs are present (see Results).

#### The Bayes/non-Bayes compromise.

From a Bayesian viewpoint, the BF provides *the* measure of the strength of evidence for genotype–phenotype association. That is, if one accepts our prior distributions and modeling assumptions, then the BF is all that is necessary to decide whether a genuine association is present. However, given the potential for debate over prior distributions, and for deviations from modeling assumptions, it is helpful to note that a *p-*value for testing *H*_0_ can be obtained from a BF through permutation. Specifically, one can compute the BF for the observed data, and for artificial data sets created by permuting observed phenotypes among cohort individuals, and obtain a *p-*value as the proportion of permuted data sets for which the BF exceeds the BF for the observed data. Being based on permutation, the resulting *p-*value is valid *irrespective of whether the model or priors are appropriate*. This *p-*value also provides a helpful way to compare our approach with standard tests of association, and, as we show below, tests based on BF appear to perform well in a wide variety of situations. Using BFs as test statistics to obtain *p-*value is referred to as the “Bayes/non-Bayes compromise” by Good [17].

#### Explaining and interpreting associations.

To “explain” observed associations we compute posterior distributions for SNP effects (*a_j_* + *d_j_* and 2*a_j_* for the heterozygote and minor-allele homozygote, respectively), with particular focus on the posterior probability that each SNP is a QTN *P*(*a_j_* ≠ 0). Here, our Bayesian regression approach has an important qualitative advantage over standard multiple regression. Specifically, if a genetic region contains multiple highly correlated SNPs, each highly correlated with the phenotype, then the correct conclusion would be any of these SNPs could be causal, without identifying which one. This will be reflected in the posterior distribution of the effects: the overall probability that at least one SNP is a QTN will be high, but (at least in the simplest case where we assume at most one QTN) this probability will be spread out over the multiple correlated SNPs. In contrast, if multiple highly correlated SNPs are included in a standard multiple regression it is possible that no one of them will produce a significant *p-*value.

We also argue that the imputation-based approach brings us closer to being able to interpret estimated effects for each SNP as actual *causal effects,* rather than simply associations. Indeed, the key to making the leap from association to causality is controlling for all potential confounding factors, and by imputing genotypes at nearby SNPs, the imputation-based approach controls for one important set of confounding factors (the nearby SNPs), which would otherwise be ignored. Thus, while functional studies provide the ultimate route to convincingly demonstrating causal effects, our approach may help target such studies on the most plausible candidate SNPs.

### Imputing genotypes

In the tagSNP design, observed genotypes G*_obs_* consist of panel genotypes at all SNPs and cohort genotypes at tagSNPs only. To apply our methods in this situation, we use sampling-based algorithms (PHASE [10,11], or fastPHASE [5]) to generate multiple imputations for the complete genotype data (all individuals at all SNPs) by sampling from 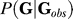 . We then incorporate these imputations into our inference: for prior *D*_1_, this involves adding a step in the MCMC scheme to sample the imputed genotypes from their posterior distribution given all the data; for prior *D*_2_ it involves simply averaging relevant calculations over imputations. Details are given in Protocols S1 and S2.

### Availability of Software

Methods described here are implemented in a software package, Bim-Bam (Bayesian IMputation-Based Association Mapping), available from the Stephens Lab website http://stephenslab.uchicago.edu/software.html.

## Results

### “Power” and Comparisons with Other Approaches

We compared the power of our approach to other common approaches via simulation. We simulated genotype and phenotype data (with μ = 0 and τ = 1) for genetic regions of length 20 kb containing a single QTN, and genetic regions of length 80 kb containing four QTNs, as follows:

(1) Using a coalescent-based simulation program, *msHOT* [18], simulate 600 haplotypes from a constant-sized random mating population, under an “infinite sites” mutation model, with (population-scaled) mutation rate θ = 0.4/kb and “background” recombination rate ρ = 0.8/kb, and a recombination hotspot (width 1 kb; recombination rate 50ρ per kb) in the center of the region.

(2) Form genotypes for a “panel” of 100 individuals by randomly pairing 200 haplotypes, and a “cohort” of 200 individuals by randomly pairing the other 400 haplotypes.

(3) Select tag SNPs from the panel data using the approach of Carlson et al. [19] with an *r* ^2^ cutoff of 0.8. As in Carlson et al. [19], SNPs with panel minor allele frequency (MAF) <0.1 were not tagged.

(4) Select which SNPs are QTNs, and their effect sizes, and simulate phenotype data for each cohort individual according to Equation 1. We considered four scenarios: (A) a “common” (MAF>0.1) QTN, with a range of effect sizes *a* = 0.2, 0.3, 0.4, 0.5 and “mild” dominance for the minor allele (*d* = 0.4*a*); (B) a common QTN, with *a* = 0.3 and “strong” dominance for the major allele (*d* = −*a*); (C) a “rare” (MAF 0.01 − 0.05) QTN, with *a* = 1 and no dominance (*d* = 0); (D) four common, relatively uncorrelated, QTNs, each with *a* = 0.3 and *d* = 0.4*a*. In each situation, we randomly chose a QTN satisfying the relevant MAF requirements (in the 600 sampled haplotypes), except under scenario (D) we first chose four tag SNP “bins” at random and then randomly chose a QTN satisfying the MAF requirement in each bin, thereby ensuring the four QTNs were relatively uncorrelated. (While real data may contain multiple highly correlated QTNs, we did not explicitly consider this case, since their effect would be similar to a single QTN.)

We compared power of tests based on the BF (under prior *D*_2_, allowing at most one QTN, using Equation 6 with four other significance tests:

(1) Two tests based on *p_min_,* the minimum *p-*value obtained from testing each SNP individually (via standard ANOVA-based methods) for association with the phenotype. These two tests differed in whether the single SNP *p-*values were obtained using the 1 degree-of-freedom (df) “allelic” test, which assumes an additive model where the mean phenotype of heterozygotes lies midway between the two homozygotes (equivalent to linear regression of phenotype on genotype), or the 2 df “genotype” test, which treats the mean of the heterozygotes as a free parameter.

(2) A test based on *p_reg_,* the global *p-*value obtained from linear regression of phenotype on all SNP genotypes (using the standard *F* statistic, coding the genotypes as 0,1 and 2 at each SNP, and assuming additivity across SNPs). See Chapman et al. [20] for example.

(3) A test based on BF*_max_*, the *maximum* single-SNP BF. We included this test for comparison with the *mean* single-SNP BF (Equation 6), to examine whether averaging information across SNPs in Equation 6 improved power.

**Figure 1:**
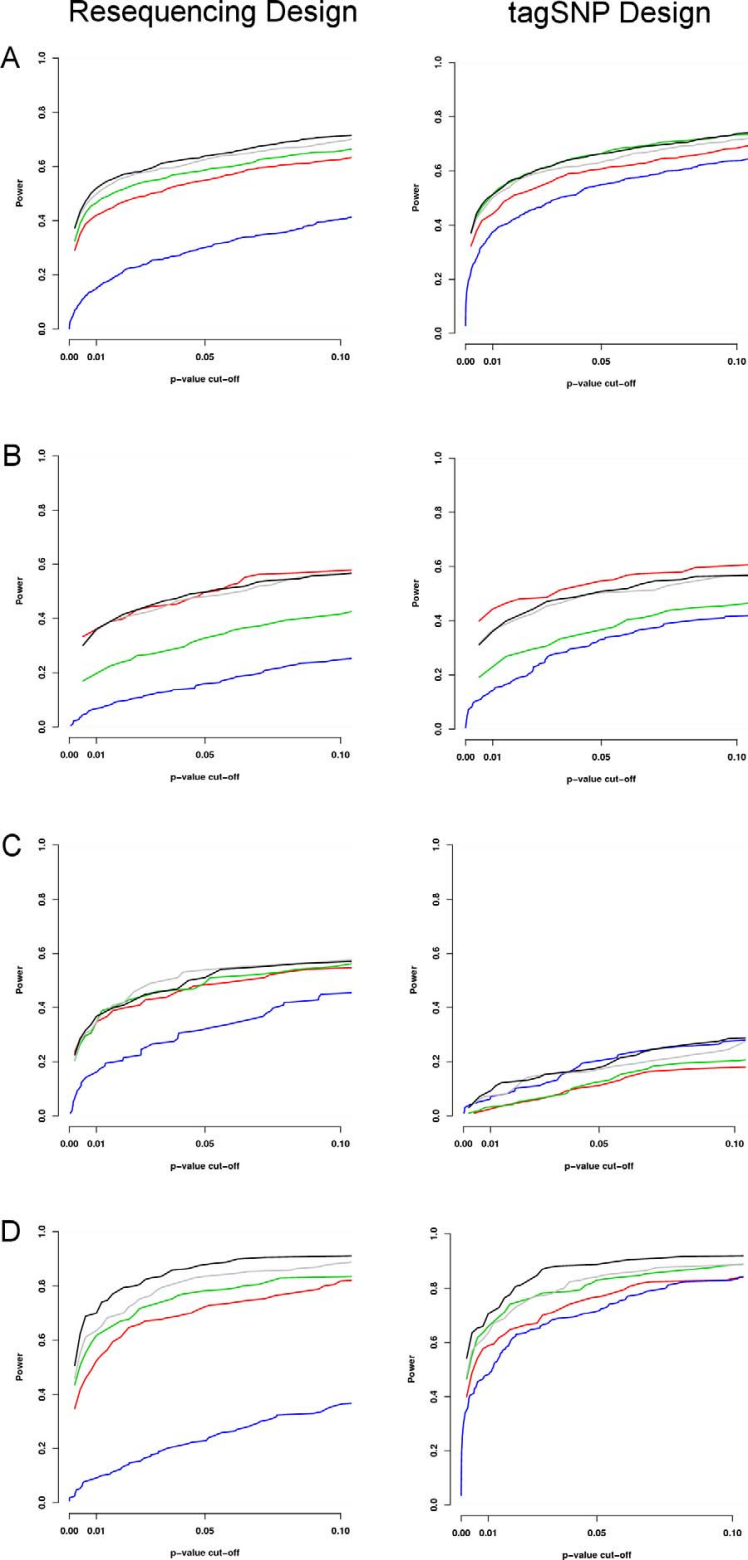
Power Comparisons (A) single common variant, modest dominance; (B) single common variant, strong dominance for minor allele; (C) single rare variant, no dominance; (D) multiple common variants. Each colored line shows power of test varying with significance threshold (type I error). Black: BF from our method (prior *D*_2_); Green: *p_min_* (allelic test); Red: *p_min_* (genotype test); Blue: *p_reg_,* multiple regression; Grey: BF*_max_*. Each column of figures shows results for data analyzed under the “resequencing design” (left) and the “tag SNP design” (right). Each row shows results for the four different simulation scenarios.

For each test, we analyzed each dataset in two ways: as if data had been collected using (i) a “resequencing design” (i.e., all individuals were completely resequenced, so genotype data are available at all SNPs in all individuals); and (ii) a “tag SNP design” (i.e., in panel individuals genotype data are available at all SNPs, but in cohort individuals genotype data are available at tag SNPs only). For the tag SNP design, we assumed haplotypic phase is known in the panel (as it is, mostly, for the HapMap data for example), but not in the cohort; however our approach can also deal with unknown phase in the panel. For *p_reg_* and *p_min_*, tests were performed on all SNPs for the resequencing design, and on tag SNPs only for the tag SNP design. For BF and BF*_max_,* single-SNP BFs were computed for all SNPs in both designs (averaging over imputed genotypes for non-tag SNPs in the tag SNP design). For *p_reg_,* we computed a *p*-value assessing significance using the standard asymptotic distribution for the *F* statistic; for the other tests we found *p*-values by permutation, using 200–500 random permutations of phenotypes assigned to cohort individuals. (The relatively small number of permutations limits the size of the smallest possible *p*-value, causing discontinuities near the origin in Figure 1).

Figure 1 shows power of each test versus type I error under both resequencing and tag SNP designs. For Scenario (A) (a single common QTN), the relative performances of methods were similar for all four effect sizes examined (unpublished data), and so we pooled these results in the figure.

Comparing *p_min_* and *p_reg_*, the single-SNP tests (*p_min_*) were more powerful when all variants (including the causal variant) were typed, or when the QTN was a common SNP and therefore “tagged” by a tag SNP, while the regression-based approach (*p_reg_*) was more powerful when the QTN was a rare SNP not “tagged” by any tag SNP. Among the two single-SNP tests, the 1 df allelic test performed as well as, or better than, the 2 df genotypic test, except in Scenario (B), where the major allele exhibits strong dominance. In particular, for Scenario (A), where the causal variant exhibits dominance, the allelic test (which assumes no dominance), performed better than the genotypic test. This is presumably because, with the effect and sample sizes considered, the extra parameter estimated in the genotypic test does not sufficiently improve model fit. Although relative performance of *p_min_* and *p_reg_* in the tag SNP design could depend on tag SNP selection scheme (and the one we used, based on pairwise LD, would seem to favor *p_min_*), it seems reasonable to expect single-SNP tests to be effective at detecting “direct” associations between the phenotype and a causal variant, or “near-direct” association between a SNP that tags a causal variant, and the regression-based approach to be better at detecting indirect associations between a phenotype and a variant not “tagged” by a single SNP (the intuition, from Chapman et al. [20], is that such variants can be highly correlated with linear combinations of tag SNPs, and thus be detected by linear regression). In principle, *p_reg_* could also effectively capture “direct” associations, but our empirical results suggest that it is less effective at this than the single SNP tests. (However, poor performance of *p_reg_* under the resequencing design may be due in part to inadequacy of the asymptotic theory when large numbers of correlated covariates are used. This might be alleviated by assessing significance of *p_reg_* by permutation.)

Turning now to our approach, except for Scenario (B) in the tag SNP design, the test based on the BF is as powerful or more powerful than the other tests. Thus, unlike *p_reg_* and *p_min_,* the BF performs well in detecting both “direct” and “indirect” associations: if the QTN is typed, the BF detects it using observed genotype data at that SNP; otherwise, it detects it using the imputed genotype data at the QTN. In Scenario (B), where the major allele exhibits strong dominance, our approach suffered slightly in power compared with the genotypic test, presumably because our prior places relatively low weight on strong dominance. However, the power loss was small compared with that of the allelic test. Thus our prior “allows” for dominance without suffering the full penalty incurred by the extra parameter in the genotypic test when dominance is less strong (Scenario [A]).

In Scenario (D), which involved multiple QTNs, tests based on the BF clearly outperformed other tests considered, even though the BF was computed allowing at most one QTN. Our explanation is that the BF, being the *average* of single-SNP BFs, has greater opportunity to capture the presence of multiple QTNs than does the minimum *p-*value. This explanation is supported by the fact that the maximum BF, BF*_max_,* performs less well than BF. To examine whether power might be further increased by explicitly allowing for multiple QTNs, we compared power for BFs computed using 1-QTN and 2-QTN models (in the 2-QTN model *p*(*l* = 1) = *p*(*l* = 2) = 0.5). We found little difference in power, although BFs for the 2-QTN model tended to be larger than BFs for the 1-QTN model, so allowing for multiple QTNs may help if the BF itself, rather than a *p*-value based on the BF, is used to measure the strength of evidence for association. In addition, considering multiple-QTN models should have advantages when attempting to *explain* an association (see below).

A second, and perhaps more surprising, situation where the BF outperforms other methods is when all SNPs are typed and tested (i.e., Scenario (A), resequencing design). Here, in contrast to Scenario (D), BF*_max_* performs similarly to the standard BF, suggesting that the power gain is due not to averaging, but to an intrinsic property of single-SNP BFs that makes them better measures of evidence than single-SNP *p*-values. Our explanation is that the BF tends to be less influenced by less informative SNPs (e.g., those with very small MAF, of which there are many in the resequencing design), whereas *p*-values tend to give equal weight to all SNPs, regardless of information content. Specifically, BFs for relatively uninformative SNPs will always lie close to 1, and should not greatly influence either the maximum or the average of the single-SNP BFs (or, more precisely, will not greatly influence differences in these test statistics among permutations of phenotypes). In contrast, *p*-values for each SNP are forced, by definition, to have a uniform distribution under *H*_0_, and so *p*-values from a large number of uninformative SNPs unassociated with the phenotype could swamp any signal generated by a single informative SNP associated with the phenotype. Although the resequencing design is currently uncommon, this observation suggests that it may generally be preferable to rank SNPs according to their BFs, rather than by *p*-values (e.g., in genome scans). It also highlights a general (rarely considered, and perhaps underappreciated) drawback of *p*-values as a measure of evidence: the strength of evidence of a given *p*-value depends on the informativeness of the test being performed, or, more specifically, on the distribution on the *p*-values under the alternative hypothesis, which is generally not known. Thus, for example, a *p*-value of 10^−5^ in a study involving few individuals may be less impressive than the same *p*-value in a larger study. In contrast, the interpretation of a BF does not depend on study size or similar factors.

### Resequencing versus Tag SNP Designs

**Figure 2:**
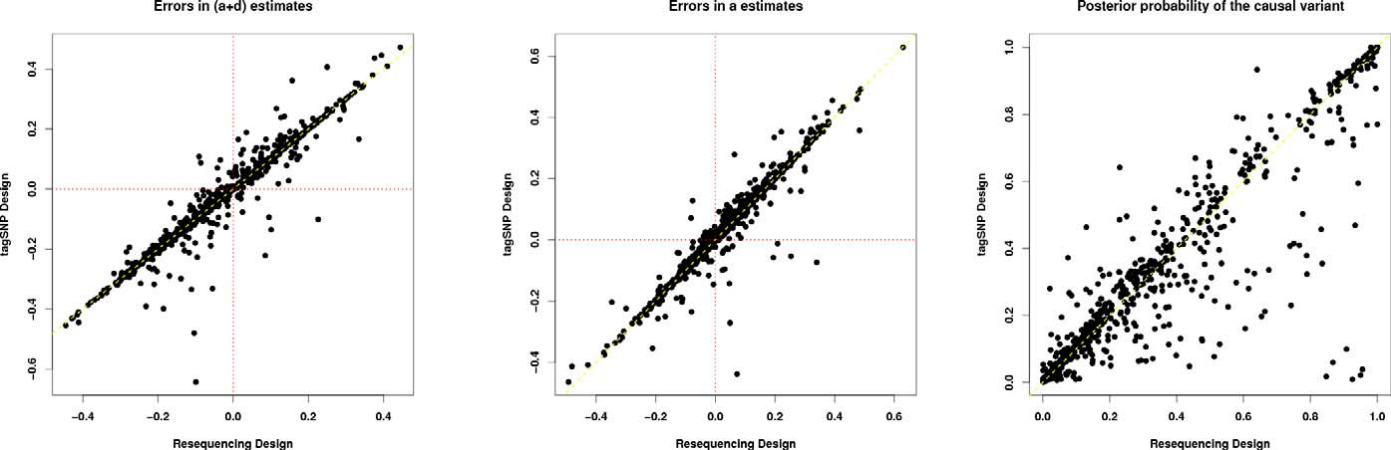
Comparison of Results for Resequencing Design (x-axis) and Tag SNP Design (y-axis) Panels show: (a) errors in the estimates (posterior means) of the heterozygote effect (*a* + *d*); (b) errors in the estimates (posterior means) of the main effect (*a*); and (c) posterior probability of being a QTN (*P*((*a, d*) ≠ (0, 0))) assigned to the causal variant.

An important feature of Figure 1 is that, for Scenarios (A), (B), and (D), where the causal SNPs are common, power is similar for the resequencing and tag SNP designs. Indeed, in these cases most other aspects of inference are also similar. For example, Figure 2 shows that, under Scenarios (A) and (B), estimated effect sizes, BFs, and posterior probability that the actual causal variant is a QTN, are typically similar for both designs. Thus under these scenarios, our imputation-based approach *effectively recreates results that would have been obtained by resequencing all individuals*.

In contrast, when the causal variant is rare, there is a noticeable drop in power for the tag SNP design versus the resequencing design, and the BFs, posterior probabilities, and effect size estimates under the two designs often differ substantially (unpublished data). This may seem slightly disappointing: one might have hoped that, even with tag SNPs chosen to capture common variants, they might also capture some rare variants. Indeed, this can happen: in some simulated data sets the rare causal variant was clearly identified by our approach, presumably because it was highly correlated with a particular haplotype background, and could thus be accurately predicted by tag SNPs. However, this occurred relatively rarely (just a few simulations out of 100).

**Figure 3:**
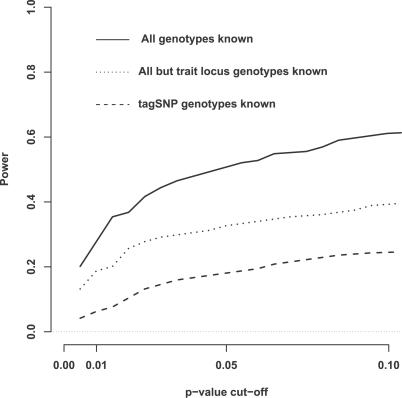
Examination of Potential Effect of Different Tag SNP Strategies on Power, When the Causal Variant is Rare (0.01 < MAF < 0.05) Solid line: Resequencing design; dashed line: tag SNP design, with tags selected using method from [19]; and dotted line: tag SNP design, with all SNPs except the causal SNP as tags.

We wondered whether a different tagging strategy, aimed at capturing rare variants, might improve performance when the causal variant is rare. The development of such strategies lies outside the scope of this paper, but, to assess potential gains that *might* be achieved, we analyzed rare-variant simulations assuming that all SNPs *except the causal variant* were typed in the cohort. Power from this approach (Figure 3) gives a conservative upper bound on what could be achieved using a more effective tagging design, without actually typing the causal variant. Although power was higher than with the *r* ^2^-based tag SNP selection, it remained substantially lower than in the resequencing design, where the causal variant is typed.

**Figure 4:**
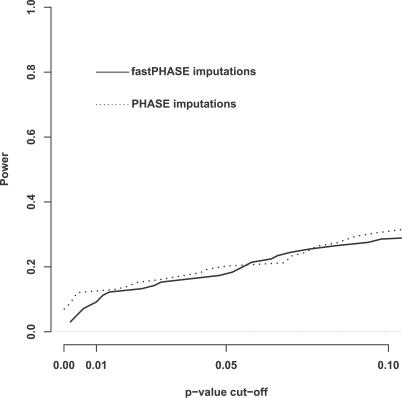
Power of the Multipoint Approach in the Rare Variant Scenario for Two Different Imputation Algorithms

We also wondered whether a different approach to impute missing genotypes (in the cohort at non-tag SNPs) might improve performance. For results above, we used the software fastPHASE [5] to impute the genotypes, so we re-ran the analysis using a different imputation algorithm [10,11]. Results for these two approaches (Figure 4) show little difference in terms of power, consistent with previous results [5] suggesting the two approaches have similar accuracy in imputing missing genotypes.

In summary, imputation-based methods appear to increase power of the tag SNP design to detect rare variants, but nevertheless remain notably less powerful than BFs based on the complete resequencing data.

### Comparison of Prior *D*_1_ and *D*_2_

**Figure 5:**
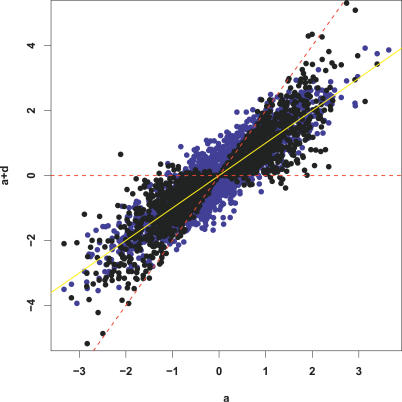
Scatter Plot of Samples from Prior Distribution of *a* (x-axis) and *a* + *d* (y-axis), for Priors *D*_1_ (Black) and *D*_2_ (Blue) The solid yellow line corresponds to *d* = 0 (additivity). The dashed red lines are the limits above and below which a SNP exhibits over-dominance.

**Figure 6:**
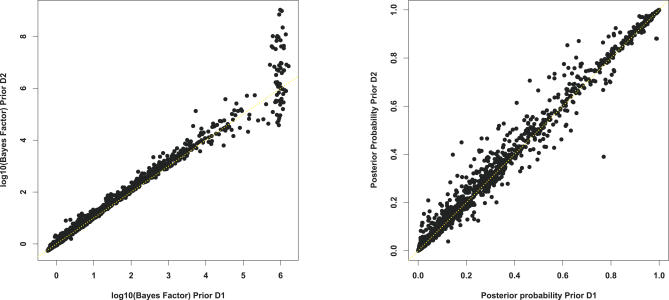
Comparison of Inferences using Prior *D*_1_ and *D*_2_ for the BF (Left) and the Posterior Probability Assigned to the Causal Locus Being a QTN (Right) Results shown are for all datasets for the common variant Scenario (A) and (B) and for both the resequencing design and the tag SNP design. The discrepancy between the larger estimated BFs is caused by the fact that we used insufficient MCMC iterations to accurately estimate very large BFs (>10^6^) under prior *D*_1_.

Priors *D*_1_ and *D*_2_ differ in their assumed correlation between the dominance effect (*d = ak*) and main effect *a*: in *D*_1_ the prior probability of overdominance is independent of *a*, whereas under *D*_2_ overdominance is more likely for small *a* than for large *a* (Figure 5). In this respect, *D*_1_ is perhaps more sensible than *D*_2_; however, *D*_2_ is computationally much simpler. To examine the effects of these priors on inference, we compared (i) the BF and (ii) the posterior probability assigned to the actual causal variant under each prior for the datasets from Scenarios (A) and (B). Results agreed quite closely (Figure 6), suggesting prior *D*_2_ provides a reasonable approximation to prior *D*_1_ in the scenarios considered. This is important, since prior *D*_2_ is computationally practical for computing BFs for very large datasets (e.g., genome-wide association studies with hundreds of thousands of SNPs), for which sampling posterior distributions of parameters using an MCMC scheme would be computationally daunting.

### Allowing for Multiple Causal Variants

When analyzing a candidate region, one would ideally like not only to detect any association, but also to identify the causal variants (QTNs). Since a candidate region could contain multiple QTNs, we implemented an MCMC scheme (using prior *D*_1_) to fit multi-QTN models where the number of QTNs is estimated from the data; here, we consider a multi-QTN model with equal prior probabilities on 1, 2, 3, or 4 QTNs. (A similar MCMC scheme could also be implemented for prior *D*_2_, and could exploit the analytical advantages of this prior to reduce computation. Indeed, for regions containing a modest number of SNPs it would be possible to examine all subsets of SNPs, and entirely avoid MCMC.)

**Figure 7:**
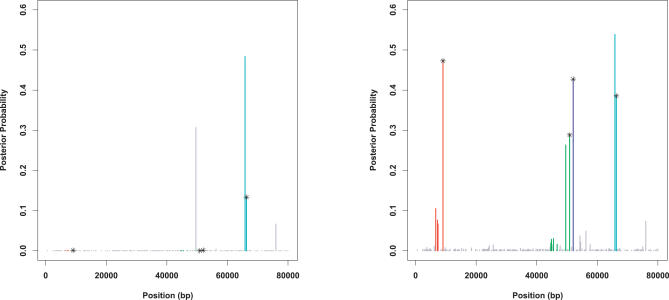
Illustration of How a Multi-QTN Model Can Provide Fuller Explanations Than a One-QTN Model for Observed Associations The figure shows, for each SNP in a dataset simulated under Scenario (D), the estimated posterior probability that it is a QTN, conditional on an association being observed. Left: Results from one-QTN model. Right: Results from multi-QTN model allowing up to four QTNs. The four actual QTNs are indicated with a star. Colors of the vertical lines indicate tag SNP “bins” (i.e., groups of SNPs tagged by the same variant).

We compare this multi-QTN model with a one-QTN model on a dataset simulated with four QTNs (scenario [D]). The estimated BF for a one-QTN model was ∼6,000, while for the multi-QTN model it was >10^5^ (we did not perform sufficient iterations to estimate how much bigger than 10^5^). Thus, if a region contains multiple causal variants, then allowing for this possibility may provide substantially higher BFs. Figure 7 shows the *marginal* posterior probabilities for each SNP being a QTN, under the one-QTN and multi-QTN models, conditional on at least one SNP in the region being a QTN. (Summarising the more complex information on posterior probabilities for *combinations* of SNPs is an important future challenge.) Under the one-QTN model, only one of the four causal SNPs has a large marginal posterior probability, whereas under the multi-QTN model all four are moderately large. Of course, other SNPs correlated with the four QTNs were also associated with the phenotype, and so have elevated posterior probabilities. This example illustrates the potential for the multi-QTN model to provide fuller explanations for associations.

### SCN1A Polymorphism and Maximum Dose of Carbamazepine

We applied our method to data from association studies involving the *SCN1A* gene and the maximum dose of carbamazepine in epileptic patients [21,22]. For this analysis, the “panel” consisted of parents from 32 trios of European descent from the CEPH Utah collection [21] and the “cohort” consisted of 425 patients of European descent for whom the maximum dose of carbamazepine had been determined [22]. Genetic data on the trios were available for 15 polymorphisms,comprising 14 SNPs and one indel, which corresponded to snps 1–15 and indel12 in Table 2 of Weale et al. [21]. For cohort individuals, genotype data are available at four tag SNPs: snp1 (rs590478), snp5 (rs8191987), snp7 (rs3812718), and snp9 (rs2126152). These SNPS were chosen to summarize haplotype diversity at the 15 panel polymorphisms (for details, see Tate et al. [22]).

We first estimated haplotypes in 64 parents using the trio option in PHASE [23]. Since trio information allows haplotypes to be accurately determined [23] we assumed these estimated panel haplotypes were correct in subsequent analyses. We then applied our method to compute a BF for overall association between genetic data and the phenotype, and to compute, for each SNP, the posterior probability that it was a QTN. In applying our method we used PHASE to impute the genotypes in the cohort at non-tag SNPs, and performed analyses under priors *D*_1_ and *D*_2_.

BFs for priors *D*_1_ and *D*_2_ were, respectively, 3.15 and 2.33, and the corresponding *p-*values (estimated using 1,000 permutations) were 0.006 and 0.019, respectively. We also computed *p-*values using single SNP tests at tag SNPs and obtained 0.007 for the allelic test and 0.019 for the genotype test. (These are essentially the two tests performed by Tate et al. [22], who reported the smallest *p-*values uncorrected for multiple comparisons.) These BFs represent only modest evidence for an association. If one were initially even somewhat skeptical about *SCN1A* as a candidate for influencing this phenotype, one might remain somewhat skeptical after analyzing these data. For example, with a 20% prior probability on variation in *SCN1A* influencing phenotype, the posterior probability of association under either prior is <50%. (Prior probability of 0.2 gives prior odds of 0.2:(1–0.2), or 1:4; a BF of 3 then gives posterior odds of 3:4, which translates to a posterior probability of 3/7.) On the other hand, *SCN1A* might be considered a relatively good candidate for influencing response to carbamazepine, since it is the drug's direct target. And, depending on follow-up costs and potential benefits of finding a functional variant, posterior probabilities of very much <50% might be deemed worth following-up.

**Figure 8:**
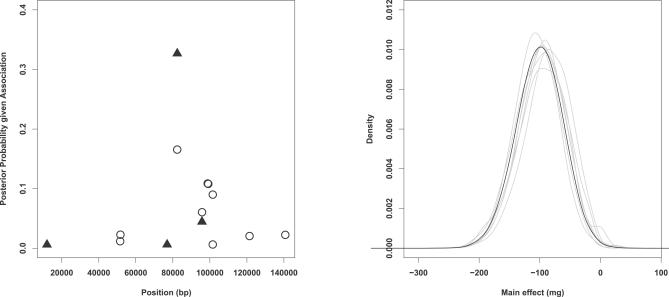
Results for the SCN1A Dataset Left panel shows the posterior probability assigned to each SNP being a QTN, with filled triangles denoting tag SNPs and open circles denoting non-tag SNPs. The right panel shows (in gray) estimated posterior densities of the additive effect for each of the seven SNPs assigned the highest posterior probabilities of non-zero effect (representing 90% of the posterior mass). The average of these curves is shown in black.

Among the 15 SNPs analyzed, snp7 was assigned the highest posterior probability of being a QTN (Figure 8). This SNP, which is a tag SNP, was also implicated by the analysis in Tate et al. [22]. However, the posterior probability of this SNP represents only 34 % of the posterior mass. Six additional SNPs are needed to encompass 90% of the posterior mass: snp6 (rs3812719), snp8 (rs490317), snp9 (rs2126152), snp10 (rs7601520), snp11 (rs2298771) and snp13 (rs7571204). The posterior distributions of the main effect, *a*, for each of these seven SNPs, conditional on it being a QTN, are very similar (Figure 8).

In summary, these data provide modest evidence of association between *SCN1A* and maximum dose of carbamazepine, and, among the SNPs analyzed, snp7 (rs3812718) appears to be the best candidate for being causal. A recent follow-up study appears to confirm this variant as being functionally important [24].

## Discussion

We described a new approach for analysis of association studies, with two important components: (i) it uses imputation of unknown genotypes, based on statistical modeling of patterns of LD, to allow untyped SNPs to be directly assessed for association with phenotype; (ii) it uses BFs, rather than *p-*values, to assess genotype–phenotype association.

The idea of trying to find associations between phenotypes and untyped variants is old, and underlies many existing methods for assessing association. In some cases this aim is implicit (e.g., testing for association between haplotypes and phenotypes can be thought of as an attempt to indirectly test untyped variants that may lie on a particular haplotype background), and in others it is explicit (to give just one example, Zöllner and Pritchard [25] place mutations on an estimated tree, and test resulting genotypes for association with phenotype). A key difference between our approach and these existing methods is that we focus on testing variants about which something is known (i.e., SNPs that are known to exist, and have documented patterns of LD), and exploiting this information. This idea, which seems in many ways more compelling than testing hypothetical untyped variants about which nothing is known, has been recently developed by several groups [5,26–31]. While there are, no doubt, multiple effective ways to implement the general strategy, attractive key features of our approach include the use of flexible statistical models for multi-locus LD to estimate missing genotypes, with uncertainty; and the use of Bayesian methods to account for uncertainty in estimated genotypes.

While several papers have suggested Bayesian approaches to association studies (e.g., [15,32,33]), our work includes some distinctive contributions. First, our prior distributions for single-SNP effects have a number of desirable properties: (i) they scale appropriately with changes in measurement units of the phenotype, (ii) they center on an additive model while allowing for dominance, and (iii) they facilitate rapid calculations. This last feature means that our work can form the foundation of simple Bayesian analyses in genome-wide association studies, e.g., computing a single-SNP BF for each SNP, as a Bayesian analogue of single-SNP hypothesis tests. This option is available in our software, but to further facilitate its use by others, and to emphasize the simplicity of the analytical calculations, we give R code for computing the BF for typed SNPs under prior *D*_2_ (see Protocol S1). A second distinctive contribution is that we compare our Bayesian approach directly with standard *p-*value based approaches, providing both qualitative insight and quantitative support for several advantages of single-SNP BFs over single-SNP *p-*values. These advantages include: (i) the BF allows for both additive and dominant effects without the additional degree of freedom incurred by the general 2 df hypothesis test; (ii) the BF better reflects the informativeness of each SNP, in particular, that SNPs with small MAF are typically less informative than SNPs with larger MAF (this advantage presumably being greatest for SNP panels containing many SNPs with small MAF); (iii) it provides a principled way to take into account prior information on each SNP, e.g., whether it lies in or near a gene whose function is believed likely to influence the trait; and (iv) averaging single-SNP BFs provides a convenient, and in some ways effective, approach to combining information across multiple SNPs in a region.

Perhaps the most important *disadvantage* of BFs compared with *p-*values is that a BF is strictly “valid” only under the assumption that both the prior and the model are “correct.” Since this is never the case in practice, BFs are never strictly valid, Our hope is to make the prior and model sufficiently accurate that resulting BFs are “useful.” (Note that *p-*values may be valid but useless: e.g., *p-*values simulated from a uniform distribution independent of phenotype and genotype data are valid, in that they are uniformly distributed under the null hypothesis, but useless.) Here, it is helpful to distinguish two different uses of BFs: as test statistics to compute permutation-based *p-*values, as in the power comparisons in this paper, and as direct measures of evidence (e.g., in “posterior odds = BF × prior odds”). Our limited experience is that *p-*values obtained from BFs are relatively robust to prior and modeling assumptions, but that the absolute values of BFs are substantially more sensitive. In particular, BFs tend to be sensitive to both (i) choice of *σ_a_, σ_d_*; and (ii) the normality assumption in the phenotype model. We now discuss each of these issues in turn.

Choice of *σ_a_, σ_d_* corresponds to quantifying prior beliefs about likely additive and dominance effect sizes. In this paper, we used (in prior *D*_2_) *σ_a_* = 0.5 and *σ_d_ = σ_a_*/2. We now believe these values are likely larger than appropriate for most studies of complex phenotypes, placing too little weight on small, but realistic, effect sizes. Our current suggested “default” procedure is to average BFs computed with *σ_a_* = 0.05, 0.1, 0.2, and 0.4, and *σ_d_ = σ_a_*/4, which places more weight on smaller effect sizes, and less weight on overdominance. We would expect to modify these values in the light of further information about typical effect sizes for particular traits. It could also be argued that, in addition to allowing a continuum of deviations from the additive model, it may make sense to specify prior probabilities for “pure” recessive or dominant models (i.e., *d = a,* −*a*). BFs under these models can be computed easily by simply replacing all heterozygous genotypes with homozygous genotypes for the major or minor allele.

Regarding the normality assumption, following a suggestion by Mathew Barber (personal communication), in practical applications, we are currently applying a normal quantile transform to phenotypes (replacing the *r*th biggest of *n* observations with the (*r* − 0.5)/*n*th quantile of the standard normal distribution) before applying our methods and computing BFs. Imposing normality on our phenotype in this way is different from the normality assumption in our phenotype model, which states that the *residuals* are normally distributed. However, in this context, where effect sizes are expected to be generally rather small, normality of phenotype and normality of residuals are somewhat similar assumptions, suggesting that this transform may be effective.

Throughout this paper, we have assumed a “population” sampling design in which phenotype and genotype data are available on a random sample from a population, and perform analyses conditional on the observed genotype data. An alternative common design involves collecting genotypes only on individuals whose phenotypes lie in the tails of the distribution [34]. To apply our methods to such designs, we suggest conditioning on *unordered* observed phenotypes, denoted {**y**}, in addition to conditioning on the genotypes G, and to perform inference for the genetic effects parameters, *β*, based on the conditional likelihood L(*β) =*P(**y** | {**y**}, *G*, *β)*. However, this conditional likelihood does not appear to be analytically tractable, and so analysis of this design may require development of computationally tractable approximations. Similarly, adapting our approach to standard case-control designs will require development of appropriate priors and computational algorithms, and represents an important area for future work.

## Supporting Information

Protocol S1 Analytical Computations for Prior D_2_
(100 KB PDF)

Protocol S2 MCMC Sampling for Prior D_1_
(66 KB PDF)

### Accession Numbers

The National Center for Biotechnology Information (NCBI) Entrez (http://www.ncbi.nlm.nih.gov/gquery/gquery.fcgi) Gene ID of the SCN1A gene is 6323.

## Abbreviations

BF: Bayes factor
df: degree of freedom
IBD: identity by descent
LD: linkage disequilibrium
MCMC: Markov Chain Monte Carlo
QTN: quantitative trait nucleotide
