# Supplementary material for: Imputation-Based Analysis of Association Studies: Candidate Regions and Quantitative Traits
Source: PLoS Genet. 2007 Jul 27;3(7):e114. doi: 10.1371/journal.pgen.0030114 (PMC1934390; doi:10.1371/journal.pgen.0030114)
Supplement: Protocol S1 — (100 KB PDF) [file pgen.0030114.sd001.pdf]

Protocol S1: Analytical computations for prior D_2

B. Servin and M. Stephens

With prior D_2, we can evaluate some posterior quantities of interest by exact computations (*i.e.* without the need for a MCMC approach). For simplicity below we focus on the model where there is a single causative SNP (SNP s say) in the model; however the case where more SNPs are included is easily handled, by extending the design matrix $\mathbf{X}$ and adding further (σ_a^2, σ_d^2) pairs to the diagonal matrix ν below.

Under prior D_2 the prior distribution for τ, β is of the form

$$\tau \sim \Gamma(\kappa/2, \lambda/2) \tag{1}$$

with density

$$p(\tau; \kappa, \lambda) = (\lambda/2)^{\kappa/2} \frac{\tau^{\kappa/2-1} \exp[-(\lambda/2)\tau]}{\Gamma(\kappa/2)}, \tag{2}$$

and

$$\beta|\tau \sim \mathcal{N}(\mathbf{0}, \nu/\tau) \tag{3}$$

where $\nu = diag(\sigma_\mu^2, \sigma_a^2, \sigma_d^2)$.

The joint posterior distribution for τ, β is available analytically as

$$\tau|\mathbf{y}, \mathbf{G} \sim \Gamma\big((n+\kappa)/2, 0.5(\mathbf{y}^t\mathbf{y} - \mathbf{B}^t\mathbf{\Omega}^{-1}\mathbf{B} + \lambda)\big) \tag{4}$$

$$\beta|\tau, \mathbf{y}, \mathbf{G} \sim \mathcal{N}(\mathbf{B}, (1/\tau)\mathbf{\Omega}) \tag{5}$$

where

$$\mathbf{B} = \mathbf{\Omega}\mathbf{X}^t\mathbf{y} \tag{6}$$

$$\mathbf{\Omega} = (\nu^{-1} + \mathbf{X}^t\mathbf{X})^{-1}. \tag{7}$$

Unlike in the main paper, we assume the first column of the design matrix $\mathbf{X}$ is a vector of 1s, to incorporate the intercept term. Thus $\mathbf{X}$ is a matrix with n rows (one for each individual), the first column containing all 1s (corresponding to an intercept term in the regression), and then two columns for each SNP included in the model, the first column being the SNP genotype (corresponding to the additive effect) and the second being a 0/1 indicator for whether the SNP genotype is a heterozygote (corresponding to the dominance effect).

We note that, in the limit $\sigma_\mu \to \infty$ and $\lambda \to 0$ the posteriors for τ, β changes appropriately with shifts and scaling operations on $\mathbf{y}$. In particular, in this limit:

1. The posterior mean for β, changes appropriately with shifts in $\mathbf{y}$. That is, adding c to each element of y will add c to the first element of $\mathbf{B}$ (the "mean" parameter, μ), leaving the other elements (the "effect" parameters, a and d) unchanged. This follows from the fact that $\mathbf{\Omega}^{-1}\mathbf{B} = \mathbf{X}^T\mathbf{y}$ implies $\mathbf{\Omega}^{-1}(\mathbf{B} + (c, 0, 0)^T) = \mathbf{X}^T(\mathbf{y} + c\mathbf{1})$, where $\mathbf{1}$ is the vector of length n whose elements are all 1s, as can be verified by straightforward algebra.

2. The posterior for τ is invariant to shifts in y (ie adding some constant c to each element of y does not change the posterior for τ), because the term $\mathbf{y}^t\mathbf{y} - \mathbf{B}^t\mathbf{\Omega}^{-1}\mathbf{B}$ does not change with shifts in y. This follows from the fact that this term is equal to $(\mathbf{y} - \mathbf{X}\mathbf{B})^T\mathbf{y}$; that $(\mathbf{y} - \mathbf{X}\mathbf{B})^T$ does not change with shifts in $\mathbf{y}$ (easily shown using 1 above); and $(\mathbf{y} - \mathbf{X}\mathbf{B})$ is orthogonal to $\mathbf{1}$, which can be checked by using the definition of $\mathbf{B}$ to show that $(\mathbf{y} - \mathbf{X}\mathbf{B})^T\mathbf{X} = \mathbf{B}^T\nu$, and then noting that the first column of $\mathbf{X}$ is $\mathbf{1}$, and the first element of $\mathbf{B}^T\nu$ is 0.

3. The posterior for τ scales appropriately with y (that is, multiplying all elements of y by some constant c essentially divides τ by c^2). This follows from elementary properties of the Gamma distribution, and because multiplying y by c has the effect of multiplying the term $\mathbf{y}^t\mathbf{y} - \mathbf{B}^t\boldsymbol{\Omega}^{-1}\mathbf{B}$ by c^2.

4. The posterior for β changes appropriately with shifts and scaling of $\mathbf{y}$. This follows from 1-3 above.

These invariance properties motivated us to use these limits ($\sigma_\mu \to \infty$ and $\lambda \to 0$) for the results in this paper. We also took $\kappa \to 0$. (In practice the results in this paper were obtained using a "large" value of σ_μ^2 although it is possible to derive the limiting results explicitly.)

As we now show, the Bayes Factor, $BF(s)$, that SNP s is a QTN vs. the "null" that no SNP is a QTN is also available analytically, and behaves sensibly in the limit $\sigma_\mu \to \infty$ and $\lambda, \kappa \to 0$.

First, integrating out β:

$$\begin{aligned} P_s(\mathbf{y}|\tau, \mathbf{G}) &= \frac{P_s(\mathbf{y}|\beta, \tau, \mathbf{G}) P_s(\beta|\tau)}{P_s(\beta|\mathbf{y}, \tau, \mathbf{G})} \\ &= (2\pi)^{-n/2} \tau^{n/2} \frac{|\boldsymbol{\Omega}|^{1/2}}{|\nu|^{1/2}} \exp\Big[-0.5(\mathbf{y}^t\mathbf{y} - \mathbf{B}^t\boldsymbol{\Omega}^{-1}\mathbf{B})\tau\Big]. \end{aligned} \tag{8}$$

Now integrating out τ:

$$\begin{aligned} P_s(\mathbf{y}|\mathbf{G}) &= \int_0^\infty P_s(\mathbf{y}|\tau, \mathbf{G}) P(\tau)\, d\tau \\ &= (2\pi)^{-n/2} \frac{|\boldsymbol{\Omega}|^{1/2}}{|\nu|^{1/2}} \int_0^\infty \tau^{(n+\kappa)/2-1} \exp\Big[-0.5(\mathbf{y}^t\mathbf{y} - \mathbf{B}^t\boldsymbol{\Omega}^{-1}\mathbf{B} + \lambda)\tau\Big] d\tau. \end{aligned} \tag{9}$$

Recognizing the above integral as the normalising constant of a $\Gamma\big((n+\kappa)/2, 0.5(\mathbf{y}^t\mathbf{y} - \mathbf{B}^t\boldsymbol{\Omega}^{-1}\mathbf{B} + \lambda)\big)$ distribution, we obtain:

$$P_s(\mathbf{y}|\mathbf{G}) = (2\pi)^{-n/2} \frac{|\boldsymbol{\Omega}|^{1/2}}{|\nu|^{1/2}} (\lambda/2)^{\kappa/2} \frac{\Gamma((n+\kappa)/2)}{\Gamma(\kappa/2)} \Big(\frac{\mathbf{y}^t\mathbf{y} - \mathbf{B}^t\boldsymbol{\Omega}^{-1}\mathbf{B} + \lambda}{2}\Big)^{-(n+\kappa)/2}. \tag{10}$$

The above expression gives the probability of the observed phenotype data under the hypothesis that SNP s is a QTN. It can also be used to obtain the probability of the phenotype data under the "null" of no effect, by setting $\nu = {\sigma_\mu}^2$, and X to be the vector of all 1s, and substituting these into (6) and (7) to compute $\mathbf{B}$ and $\boldsymbol{\Omega}$. This gives

$$P_0(\mathbf{y}) = (2\pi)^{-n/2} \frac{\Omega_0^{1/2}}{\sigma_\mu} (\lambda/2)^{\kappa/2} \frac{\Gamma((n+\kappa)/2)}{\Gamma(\kappa/2)} \Big(\frac{\mathbf{y}^t\mathbf{y} - \Omega_0 n^2 \bar{y}^2 + \lambda}{2}\Big)^{-(n+\kappa)/2} \tag{11}$$

where $\Omega_0 = ((\sigma_\mu^2)^{-1} + n)^{-1}$.

The Bayes Factor $BF(s)$ is then the ratio of (10) to (11):

$$BF(s) = \frac{P_s(\mathbf{y}|\mathbf{G})}{P_0(\mathbf{y})} = \frac{|\boldsymbol{\Omega}|^{1/2}}{\Omega_0^{1/2}} . \frac{1}{\sigma_a \sigma_d} . \Big[\frac{\mathbf{y}^t\mathbf{y} - \mathbf{B}^t\boldsymbol{\Omega}^{-1}\mathbf{B} + \lambda}{\mathbf{y}^t\mathbf{y} - \Omega_0 n^2 \bar{y}^2 + \lambda}\Big]^{-(n+\kappa)/2}. \tag{12}$$

Limiting value of the BF Our priors for β and τ are obtained by taking the limit $+\infty$ for σ_μ and 0 for both λ and κ. The limit of $BF(s)$ with respect to λ and κ is:

$$\lim_{\substack{l \to 0 \\ m \to 0}} BF(s) = \frac{|\boldsymbol{\Omega}|^{1/2}}{\Omega_0^{1/2}} . \frac{1}{\sigma_a \sigma_d} . \Big[\frac{\mathbf{y}^t\mathbf{y} - \mathbf{B}^t\boldsymbol{\Omega}^{-1}\mathbf{B}}{\mathbf{y}^t\mathbf{y} - \Omega_0 n^2 \bar{y}^2}\Big]^{-n/2} \tag{13}$$

Finally, taking the limit when $\sigma_\mu \to \infty$ is straightforward because $\boldsymbol{\Omega}$ has a finite limit as $\sigma_\mu \to \infty$.

Bayes Factor for multiple SNPs If we assume the effects of multiple SNPs are additive, the computation of the Bayes Factor, $BF(s_1, s_2, \ldots, s_p)$ that p SNPs $(s1, \ldots, s_p)$ are QTNs vs. no SNP is a QTN can be done following the same approach. $BF(s_1, s_2, \ldots, s_p)$ is computed with equation (12), after modification of the design matrix $\mathbf{X}$ and the prior matrix ν. $\mathbf{X}$ is then a $(n \times (2p+1))$ matrix: the first column of $\mathbf{X}$ is filled with 1's and each pair of column $\{(2i, 2i+1); i \in [1,p]\}$ relates the individuals with their genotypes. ν is a $((2p+1) \times (2p+1))$ diagonal matrix with $\nu_{1,1} = \sigma_\mu^2$, $\nu_{2i,2i} = \sigma_a^2$ and $\nu_{2i+1,2i+1} = \sigma_d^2$ for $i \in [1,p]$. Note that this implies that the term $\frac{1}{\sigma_a \sigma_d}$ in equation (12) becomes $\frac{1}{(\sigma_a \sigma_d)^p}$.

Bayes Factor for a region Here we show how to compute the Bayes Factor,BF, for association $(H1)$ vs no association $(H0)$. Given a prior distribution on the number of QTNs $p(l)$ on $[1..L]$, we have:

$$\mathrm{BF} = \sum_{l=1}^{L} p(l) \frac{1}{\binom{n_s}{l}} \sum_{(s1,\ldots,s_l) \in c(l,n_s)} \mathrm{BF}(s_1, \ldots, s_l) \qquad (14)$$

where $c(l, n_s)$ denotes the ensemble of all possible combinations of l SNPs taken from all n_s SNPs. In the particular case where $L = 1$, this reduces to:

$$\mathrm{BF} = (1/n_s) \sum_{s=1}^{n_s} \mathrm{BF}(s)$$

which is the mean of the single SNP Bayes Factors over the region.

R code to compute BF for prior D_2

The following R code computes the $\log_{10}(\mathrm{BF})$ for a single-SNP, whose genotypes (coded as 0, 1 or 2 copies of the minor allele) are contained in the vector `g`, given a corresponding vector of phenotypes `y`, and user-supplied values for σ_a and σ_d. (Individuals with missing data in either `g` or `y` are ignored in this calculation.)

```
logBF = function(g,y,sigmaa,sigmad){
subset = complete.cases(y) & complete.cases(g)
y=y[subset]
g=g[subset]
n=length(g)
X = cbind(rep(1,n),g,g==1)
invnu = diag(c(0,1/sigmaa^2,1/sigmad^2))
invOmega = invnu + t(X) %*% X
B = solve(invOmega, t(X) %*% cbind(y))
invOmega0 = n
return(-0.5*log10(det(invOmega)) + 0.5*log10(invOmega0) - log10(sigmaa)
- log10(sigmad) -(n/2) * (log10( t(y- X %*% B) %*% y)
- log10(t(y) %*% y - n*mean(y)^2) ))
}
```
