# Supplementary material for: Imputation-Based Analysis of Association Studies: Candidate Regions and Quantitative Traits
Source: PLoS Genet. 2007 Jul 27;3(7):e114. doi: 10.1371/journal.pgen.0030114 (PMC1934390; doi:10.1371/journal.pgen.0030114)
Supplement: Protocol S2 — (66 KB PDF) [file pgen.0030114.sd002.pdf]

Protocol S2: MCMC sampling for prior D_1

B. Servin and M. Stephens

When using prior D_1, we estimate BFs and posterior distributions via MCMC. Specifically, given observed phenotypes $\mathbf{y}$ and observed genotype data $\mathbf{G}_{obs}$ (which for the tag SNP design will consist of genotypes of all SNPs in the panel, and genotypes of tag SNPs in the cohort), we sample from the joint distribution of the model parameters, $(\mu, \tau, \beta = (\mathbf{a}, \mathbf{k}))$, and of the "complete" genotypes $\mathbf{G}$ (which will consist of genotypes at all SNPs in all individuals, including particularly the genotypes at the non tag SNPs in the cohort).

In outline the approach is:

1. Update $\mathbf{G}$ given the genotype information available $\mathbf{G}_{obs}$.
2. Update the genetic effects parameters $\mathbf{a}, \mathbf{k}, \mu$
3. Update τ from $\tau|\mathbf{a}, \mathbf{k}, \mu, \mathbf{G}, \mathbf{y}$

These steps are iterated many times to obtain samples from a Markov-Chain whose stationary distribution is the joint posterior distribution of all model parameters.

Updating the genotypes To update $\mathbf{G}$ we first propose a new value $\mathbf{G}'$ from $P(\mathbf{G}|\mathbf{G}_{obs})$, and use a Metropolis-Hastings step to accept or reject it. The new configuration is accepted with probability:

$$a = min\left(1, \frac{P(\mathbf{y}|\mu, \mathbf{a}, \mathbf{k}, \tau, G')}{P(\mathbf{y}|\mu, \mathbf{a}, \mathbf{k}, \tau, G)}\right). \quad (1)$$

(Here the proposal probability has cancelled with the prior distribution to yield this acceptance probability.)

In practice, we actually generated a large number of samples from $P(\mathbf{G}|\mathbf{G}_{obs})$, using PHASE (1; 2) or fastPHASE (3) and propose new configurations by choosing uniformly at random from this sample.

Update of the genetic effects To describe this update we introduce additional notation. Let γ denote the set of SNPs which are QTNs, L denote the maximum number of QTNs allowed under our prior, and n_S denote the total number of SNPs in the region. To update the genetic effect parameters we first propose a new value γ^* for γ, as follows. With probability 0.2 we set $\gamma^* = \gamma$. Otherwise we propose a new value γ^* by adding and/or removing a SNP from γ:

1. If γ contains no SNPs, we add a new SNP at random.

2. If γ includes L SNPs: if $L = n_S$ then remove a SNP at random; otherwise with probability 0.5 remove a SNP at random, and with probability 0.5 remove a SNP at random from γ and add a randomly-chosen SNP currently not in γ.

3. In all other configurations for γ, we either change the status (*i.e.* from included to not included or from not included to included) of a SNP at random (with probability 0.5) or switch a SNP included with a non-included SNP (probability 0.5).

Then, given the proposed new set of QTNs, γ^*, we jointly propose new values for their respective regression coefficients and the reference mean μ, by sampling from the proposal distribution

$$q_{ak}(\mu^*, \mathbf{a}^*, \mathbf{k}^*|\gamma^*) \sim \mathcal{N}(\mathbf{B}, \mathbf{V}), \tag{2}$$

where $\mathbf{B} = \mathbf{V}\mathbf{X}^t\mathbf{y}$, $\mathbf{V} = (\tau\mathbf{X}^t.\mathbf{X} + \mathbf{v}^{-1})^{-1}$ and $\mathbf{v} = diag(\sigma_\mu^2, \sigma_a^2/\tau, \sigma_k^2\sigma_a^2/\tau, \dots, \sigma_a^2, \sigma_k^2\sigma_a^2/\tau)$. Here, unlike in the main paper, we assume the design matrix, $\mathbf{X}$, has the first column a vector of 1s, to incorporate the intercept term. The dimensions of $\mathbf{X}$ and $\mathbf{v}$ are function of the number of QTNs. We took σ_μ^2 to be very large.

The idea here is that q_{ak} is an approximation to the conditional distribution of $\mu, \mathbf{a}, \mathbf{k}$ given all the other parameters. Specifically, it would be the posterior distribution of the regression coefficients if priors on the additive effect and dominance effect were joint normal, with prior distribution $\hat{p}(\mu, \mathbf{a}, \mathbf{k}|\gamma, \tau) \sim \mathcal{N}(\mathbf{0}, \mathbf{v})$. As a result,

$$q_{ak}(\mu, \mathbf{a}, \mathbf{k}|\gamma) = \frac{P(\mathbf{y}|\mu, \mathbf{a}, \mathbf{k}, \tau, \gamma, \mathbf{G})\hat{p}(\mu, \mathbf{a}, \mathbf{k}|\gamma, \tau)}{\hat{p}(\mathbf{y}|\gamma, \tau, \mathbf{G})} \tag{3}$$

where the denominator $\hat{p}(\mathbf{y}|\gamma, \tau, \mathbf{G})$ is the integral of the numerator over $\mu, \mathbf{a}, \mathbf{k}$, which can be computed analytically as in prior D_2 below, leading to:

$$\hat{p}(\mathbf{y}|\gamma, \tau, \mathbf{G}) = (2\pi)^{-n/2}\tau^{n/2}\frac{|\mathbf{V}|^{1/2}}{|\mathbf{v}|^{1/2}}\exp\Big[-0.5(\mathbf{y}^t\mathbf{y} - \mathbf{B}^t\mathbf{V}^{-1}\mathbf{B})\Big]. \tag{4}$$

The new proposed values are then accepted with probability:

$$\begin{aligned} a &= min\left(1, \frac{P(\mathbf{y}|\mu^*, \mathbf{a}^*, \mathbf{k}^*, \gamma^*, \tau, \mathbf{G})P(\mu^*, \mathbf{a}^*, \mathbf{k}^*|\gamma^*, \tau)P(\gamma^*)}{P(\mathbf{y}|\mu, \mathbf{a}, \mathbf{k}, \gamma, \tau, \mathbf{G})P(\mu, \mathbf{a}, \mathbf{k}|\gamma, \tau)P(\gamma)} \frac{q_{ak}(\mu, \mathbf{a}, \mathbf{k}|\gamma)}{q_{ak}(\mu^*, \mathbf{a}^*, \mathbf{k}^*|\gamma^*)} \frac{q(\gamma|\gamma^*)}{q(\gamma^*|\gamma)}\right) \\ &= min\left(1, \frac{\hat{p}(\mathbf{y}|\gamma^*, \tau, \mathbf{G})P(\mu^*, \mathbf{a}^*, \mathbf{k}^*|\gamma^*, \tau)}{\hat{p}(\mathbf{y}|\gamma, \tau, \mathbf{G})\hat{p}(\mu^*, \mathbf{a}^*, \mathbf{k}^*|\gamma^*, \tau)} \frac{\hat{p}(\mu, \mathbf{a}, \mathbf{k}|\gamma, \tau)}{P(\mu, \mathbf{a}, \mathbf{k}|\gamma, \tau)} \frac{P(\gamma^*)}{P(\gamma)} \frac{q(\gamma|\gamma^*)}{q(\gamma^*|\gamma)}\right). \end{aligned} \tag{5}$$

As the effect of a QTN typically depends substantially on which other SNPs are QTNs, this joint update of all the QTNs effects at once is essential to achieve a good mixing of the chain.

Update of τ We update τ by sampling from its full conditional distribution:

$$\tau|\mu, \beta, \mathbf{y}, \mathbf{G} \sim \Gamma\Big(n/2, (\sum_i (y_i - (\mu + \mathbf{x_i}(\mathbf{G})\beta))^2)/2\Big) \tag{6}$$

where $\mathbf{x_i}(\mathbf{G})$ is the ith row of the design matrix formed from genotypes $\mathbf{G}$.

Approximation of BFs from MCMC output To approximate the BF we applied the MCMC scheme with a prior odds of 1 (i.e. probability of 0.5 on each of the null and alternative models), and then estimate the BF for the alternative vs the null model using the estimated posterior odds, being the ratio of the number of iterations in which γ contains at least one SNP to the number of iterations in which γ contains no SNPs (adding one to both the numerator and denominator to deal with potential 0 counts).

References

[1] Stephens M, Smith N, Donnelly P (2001) A new statistical method for haplotype reconstruction from population data. American Journal of Human Genetics 68: 978–989.

[2] Stephens M, Scheet P (2005) Accounting for decay of linkage disequilibrium in haplotype inference and missing-data imputation. Am J Hum Genet 76: 449–62.

[3] Scheet P, Stephens M (2006) A fast and flexible statistical model for large-scale population genotype data: applications to inferring missing genotypes and haplotypic phase. Am J Hum Genet 78: 629–44.
